# Oxidative stress index as a potent predictor of disease severity and inflammatory burden in hospitalized COVID-19 patients

**DOI:** 10.1016/j.imj.2026.100261

**Published:** 2026-05-07

**Authors:** Abdisa Tufa Bedada, Tewodros Haile Gebremariam, Tsegahun Manyazewal, Yidnekachew Asrat Birhan, Tesfaye Tolessa Dugul, Solomon Genet Gebre, Dominic-Luc Webb, Per Martin Hellström

**Affiliations:** a Department of Medical Biochemistry, School of Biomedicine, College of Health Sciences, Addis Ababa University, Addis Ababa 9086, Ethiopia; b Department of Internal Medicine, School of Medicine, College of Health Sciences, Addis Ababa University, Addis Ababa 9086, Ethiopia; c Centre for Innovative Drug Development and Therapeutic Trials for Africa (CDT-Africa), College of Health Sciences, Addis Ababa University, Addis Ababa 9086, Ethiopia; d Department of Physiology, College of Health and Allied Sciences, St Joseph University, Dar es Salaam 11007, Tanzania; e DLW Bioanalytics, Märsta SE-19533, Sweden; f Gastroenterology and Hepatology Unit, Department of Medical Sciences, Uppsala University, Uppsala 256SE-75105, Sweden

**Keywords:** SARS-CoV-2, Oxidative stress, Biomarker, Inflammation, Cytokine storm

## Abstract

**Background:**

While the acute phase of the COVID-19 pandemic has passed, understanding its pathophysiology remains critical for managing long-term sequelae and future viral threats. Oxidative stress is a proposed key driver of COVID-19 severity, but its relationship with the dynamic inflammatory cascade and its value as a standalone biomarker requires further validation.

**Methods:**

Case-control study was conducted to quantify systemic oxidative stress burden in 109 hospitalized COVID-19 patients (56 severe, 53 mild-moderate) and 112 healthy controls. Serum total oxidant status (TOS) and total antioxidant status (TAS) were measured, and the oxidative stress index (OSI) was calculated and correlated to the inflammatory mediators (IL-6, IL-8, IL-10, IP-10, CRP, SAA).

**Results:**

COVID-19 patients exhibited a profound redox imbalance, characterized by elevated TOS and OSI, and depleted TAS compared to controls (*p* < 0.05). This imbalance was exacerbated in severe disease, where TOS and OSI were significantly increased. The OSI demonstrated superior diagnostic performance for disease severity (AUC = 0.90; 95% CI: 0.86–0.95) compared to TOS (AUC = 0.89) and TAS (AUC = 0.78). Crucially, both TOS and OSI showed strong positive correlations with inflammatory cytokines and acute-phase proteins (IL-6, IL-10, IP-10, CRP, SAA; all *p* < 0.001). After adjusting for age, time from symptom onset, and comorbidity burden (Charlson Comorbidity Index), multivariate analysis confirmed that high OSI was independently associated with disease severity (adjusted OR: 4.8; 95% CI: 1.9–12.1).

**Conclusions:**

The OSI is a robust, integrative biomarker that strongly reflects COVID-19 severity and is closely linked to the hyperinflammatory state. The OSI is strongly correlated with increased inflammatory cytokines and acute-phase proteins in acute COVID-19.

## Highlights

• COVID-19 causes a profound redox imbalance characterized by elevated total oxidant status and depleted antioxidant status, translating to an increased oxidative index.
• A severity-related increase follows the COVID-19 infection in the oxidative index.
• The COVID-19-induced increase in the oxidative index is strongly correlated with increased inflammatory cytokines and acute-phase proteins.

## Graphical abstract

**Figure:**
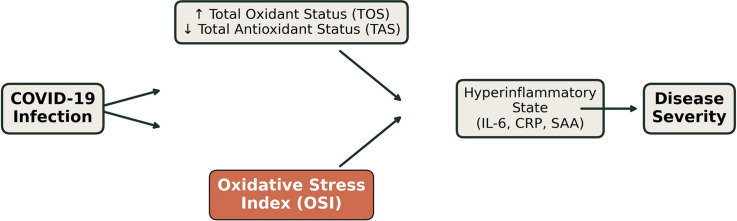

## 1 Introduction

The global crisis instigated by severe acute respiratory syndrome coronavirus 2 (SARS-CoV-2) revealed critical gaps in our understanding of virus-induced host damage. While acute respiratory distress syndrome and multi-organ failure were hallmarks of severe coronavirus disease 2019 (COVID-19), the underlying mechanisms driving this disproportionate host response are still being elucidated.^1^ A central hypothesis implicates a maladaptive interplay between rampant inflammation and severe oxidative stress.^2^^,^^3^

Oxidative stress, resulting from an imbalance between reactive oxygen species (ROS) and antioxidant defenses, is a common pathway in the pathogenesis of many viral infections.^4^ In COVID-19, viral entry via the ACE2 receptor can directly disrupt mitochondrial function, leading to ROS overproduction.^5^ This oxidative burst activates the NLRP3 (NOD-, LRR- and pyrin domain-containing protein 3) inflammasome and NF-κB signaling pathways, fueling a vicious cycle of pro-inflammatory cytokine release (“cytokine storm”) that further amplifies ROS generation from activated immune cells.^6^^,^^7^ This synergistic relationship between oxidation and inflammation is considered a primary driver of endothelial damage, thromboembolic complications, and ultimately, organ dysfunction.^8^

Despite this compelling theoretical framework, direct and comprehensive clinical evidence linking quantified oxidative stress to disease severity and specific inflammatory mediators in COVID-19 remains limited. Many early studies^9^, ^10^, ^11^ were constrained by small sample sizes or a narrow focus on individual oxidative markers.

This study aimed to perform a comprehensive comparative analysis of the systemic oxidative stress profile in hospitalized COVID-19 patients. We quantified the total oxidant status (TOS), total antioxidant status (TAS), and the derived oxidative stress index (OSI) to capture the net redox balance. Our primary objective was to evaluate the utility of these parameters as biomarkers for distinguishing between disease severity levels. Our secondary objective was to evaluate the correlation between the magnitude of oxidative stress and the levels of key circulating inflammatory mediators, thereby providing a deeper insight into the integrated pathophysiology of severe COVID-19.

## 2 Materials and methods

### 2.1 Study design and participants

A case-control study was conducted at Tikur Anbessa Specialized Hospital, Addis Ababa, Ethiopia, from January to December 2021. The study was approved by the hospital and enrolled 109 adult patients hospitalized with laboratory-confirmed COVID-19 via real-time polymerase chain reaction . Patients were classified into mild-moderate (*n* = 53) or severe (*n* = 56) groups based on WHO clinical progression scale criteria.^12^ Mild-moderate cases presented with no hypoxia or pneumonia, or with clinical signs of pneumonia but oxygen saturation (SpO₂) ≥ 90%. Severe cases required intensive care due to severe pneumonia with SpO₂ < 90%. Blood samples were collected within 48 h of hospital admission, prior to the administration of immunomodulatory therapies (e.g., dexamethasone, tocilizumab). The median time from symptom onset to sampling was 7 days (IQR: 5–10 days). Patients requiring intensive care unit admission were sampled prior to or within 24 h of intensive care unit transfer. Exclusion criteria included anemia, pregnancy, and recent use of statins or antioxidant supplements. A control group of 112 healthy, SARS-CoV-2 real-time polymerase chain reaction negative adults with no chronic comorbidities was recruited.

### 2.2 Sample collection and laboratory analysis

Venous blood (5 mL) was drawn, serum was separated by centrifugation and stored at −80 °C until batch analysis. Oxidative stress parameters: TOS and TAS were measured using commercial colorimetric assay kits (Immundiagnostik AG, Bensheim, Germany). The OSI was calculated as the ratio: OSI = (TOS/TAS).^13^ Inflammatory biomarkers: Serum levels of interleukin (IL)-6, IL-8, IL-10, interferon γ-induced protein 10 kDa (IP-10), C-reactive protein (CRP), and serum amyloid A (SAA) were quantified using a multiplex electrochemiluminescence immunoassay (Mesoscale Discovery, Rockville, MD, USA). This panel was selected to capture key aspects of the COVID-19-associated hyperinflammatory response, including canonical pro-inflammatory cytokines (IL-6), chemokines involved in T-cell recruitment (IP-10), immunomodulatory cytokines (IL-10), and major acute-phase proteins (CRP, SAA).

### 2.3 Statistical analysis

Data were analyzed using SPSS v25.0 (IBM Corp, Amonk, NY, USA), GraphPad Prism v8 (San Diego, USA) and R Statistical Software v4.3.0 (Vienna, Austria). Categorical variables were compared using the Chi-square test. Continuous, non-normally distributed data (assessed by the Shapiro-Wilk test) were presented as median (interquartile range, IQR) and compared using the Mann-Whitney *U*-test. Spearman’s rank correlation was used to assess relationships between oxidative and inflammatory markers.

Missing data: No missing data were present for the primary oxidative stress parameters (TOS, TAS, and OSI) or inflammatory biomarkers.

Power and bootstrap validation: A post-hoc power analysis was performed using G*Power 3.1. With an observed area under the curve (AUC) of 0.90 for OSI, α = 0.05, and our sample size (*n* = 109), the study achieved > 99% power to detect an AUC significantly different from 0.5. To assess the stability of the receiver operating characteristic (ROC) estimates, bootstrap resampling with 1000 repetitions was performed. Bootstrap-derived 95% confidence intervals (CIs) are reported alongside conventional intervals for key metrics. Multicollinearity assessment: Variance inflation factors (VIF) were calculated for all independent variables entered into the multivariate logistic regression model to check for multicollinearity. A VIF < 5 was considered acceptable. ROC curve and diagnostic performance analysis: The diagnostic accuracy of biomarkers was determined by ROC curve analysis, reporting the AUC with 95% CI. The DeLong test was used for the statistical comparison of AUCs between different biomarkers. Diagnostic performance was further characterized by calculating sensitivity, specificity, positive predictive value, negative predictive value, positive likelihood ratio, and negative likelihood ratio at the optimal cut-off point determined by the Youden Index. Multivariate regression analysis: To identify independent predictors of disease severity (severe vs. mild-moderate), a binary logistic regression model was employed. The model was adjusted for age, time from symptom onset to sampling (as a continuous variable), and comorbidity burden quantified using the Charlson Comorbidity Index (CCI). To test for effect modification, an interaction term between OSI and age (OSI × Age) was included in a separate model. Results are presented as odds ratios (OR) with 95% CI. A two-tailed *p* < 0.05 was considered statistically significant.

## 3 Results

### 3.1 Demographic and clinical characteristics

The healthy control group was significantly younger than the patient groups (*p* < 0.001), but sex distribution was similar (Table 1). Among COVID-19 patients, 49% had at least one comorbidity. The comorbidity burden, quantified by the CCI, was significantly higher in the severe group (median CCI: 2, IQR: 1–4) compared to the mild-moderate group (median CCI: 0, IQR: 0–1; *p* < 0.001). There were no significant differences between mild-moderate and severe groups in time from symptom onset to sampling (*p* = 0.12) or prior use of antiviral agents (*p* = 0.45).

**Table 1:** Baseline characteristics of study participants.

Characteristic	Healthy controls (*n* = 112)	All COVID-19 patients (*n* = 109)	Mild-moderate COVID-19 patients (*n* = 53)	Severe COVID-19 patients (*n* = 56)	*p* (Severe vs. Mild-Moderate patients)
Age, years (median, IQR)	25 (22–32)	45 (29–65)	32 (25–51)	60 (39–66)	**< 0.001**
Male, *n* (%)	66 (59)	62 (57)	31 (58)	31 (55)	0.847
Chronic lung disease, *n* (%)	0 (0)	12 (11)	2 (4)	10 (18)	**0.029**
Charlson comorbidity index, median (IQR)	0 (0–0)	1 (0–3)	0 (0–1)	2 (1–4)	**< 0.001**
Time from symptom onset to sampling, days, median (IQR)	—	7 (5–10)	7 (5–99)	8 (5–10)	0.12
Prior use of survival agents, *n* (%)	—	28 (26)	15 (28)	13 (23)	0.45

*Abbreviations*: COVID-19, coronavirus disease 2019; IQR, interquartile range.

### 3.2 Oxidative stress parameters are dysregulated in COVID-19 and correlate with severity

**Fig. 1:**
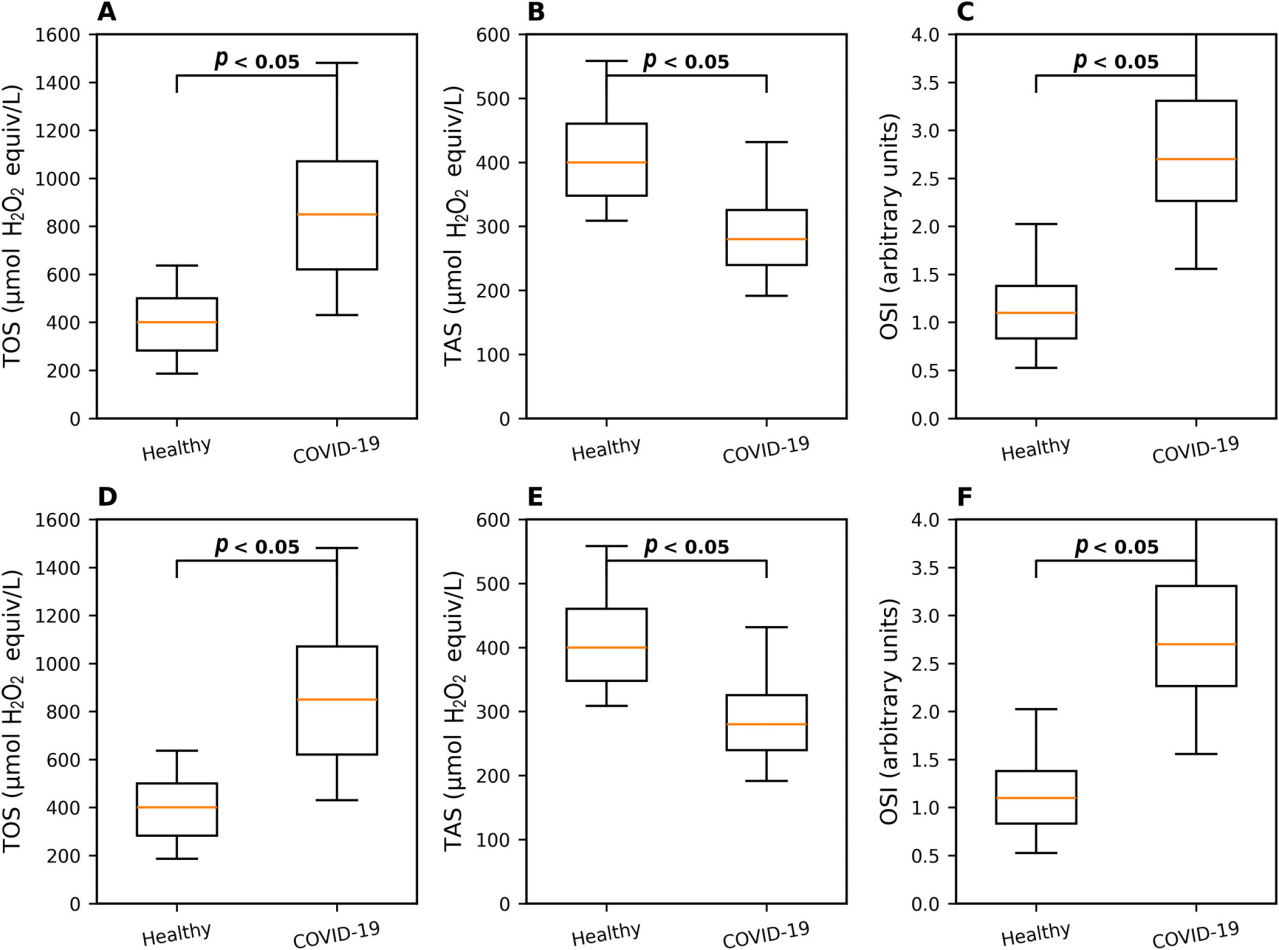
Dysregulation of oxidative stress parameters in COVID-19 patients compared to healthy controls. (A) TOS (total oxidant status), (B) TAS (total antioxidant status), and (C) OSI (oxidative stress index) in COVID-19 patients compared to healthy subjects. TAS, TOS and OSI adjusted for age (age ≤ 40 years) presented in (D), (E), and (F). Box-and-whisker plots are median, interquartile range, and minimum to maximum. Data are presented as median (interquartile range). Statistical comparisons were performed using the Mann-Whitney *U* test. *Abbreviations*: TOS, total oxidant status; TAS, total antioxidant status; OSI, oxidative stress index.

COVID-19 patients had a significantly elevated oxidative burden, evidenced by higher TOS and OSI, and lower TAS compared to healthy controls (all *p* < 0.05; Fig. 1A–C). After adjusting for age (stratifying at ≤ 40 years), these differences remained highly significant (Fig. 1D–F).

**Fig. 2:**
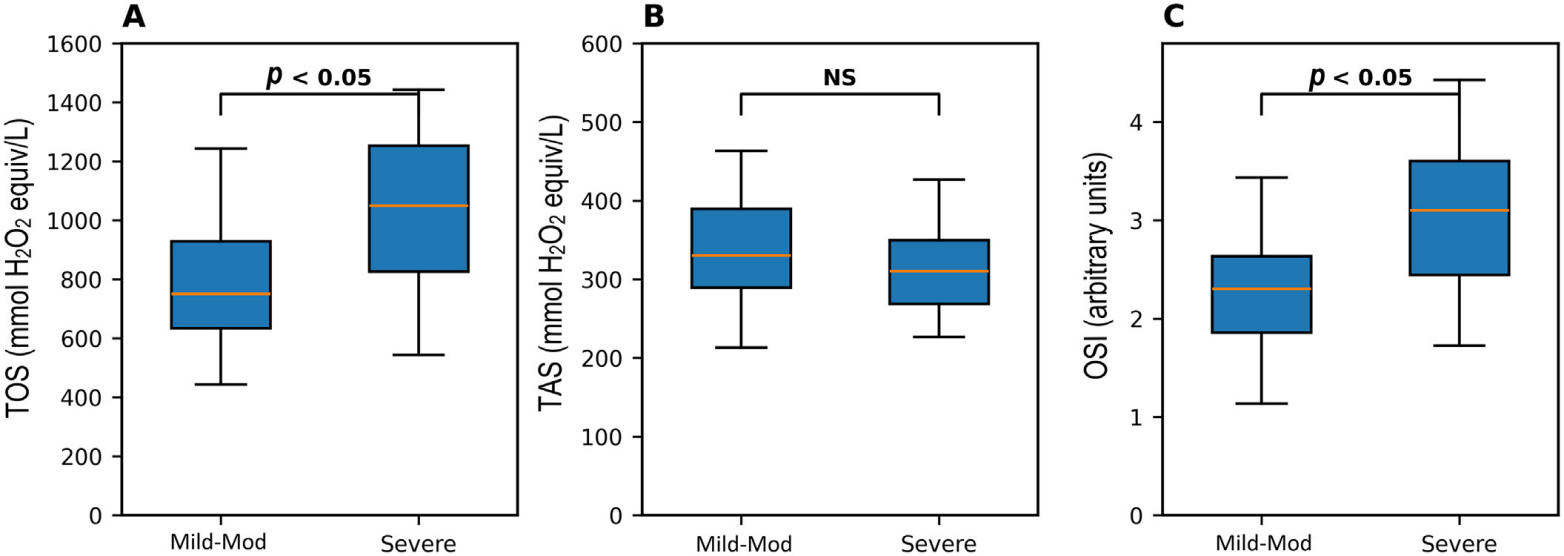
Comparison of oxidative stress parameters between mild-moderate and severe COVID-19 patients. (A) Comparison of TOS, (B) TAS, and (C) OSI between mild-to-moderate and severe COVID-19 patients. Box-and whisker plots are median, interquartile range, and minimum to maximum. Data are presented as median (interquartile range). Statistical comparisons were performed using the Mann-Whitney *U* test. “NS” indicates not statistically significant. *Abbreviations*: TOS, total oxidant status; TAS, total antioxidant status; OSI, oxidative stress index.

Critically, when comparing patient groups, TOS and OSI were significantly higher in severe cases than in mild-moderate cases (*p* < 0.05; Fig. 2A, C). TAS showed a non-significant decreasing trend with increasing severity (Fig. 2B).

### 3.3 Inflammatory biomarkers are elevated and correlated with oxidative stress

**Fig. 3:**
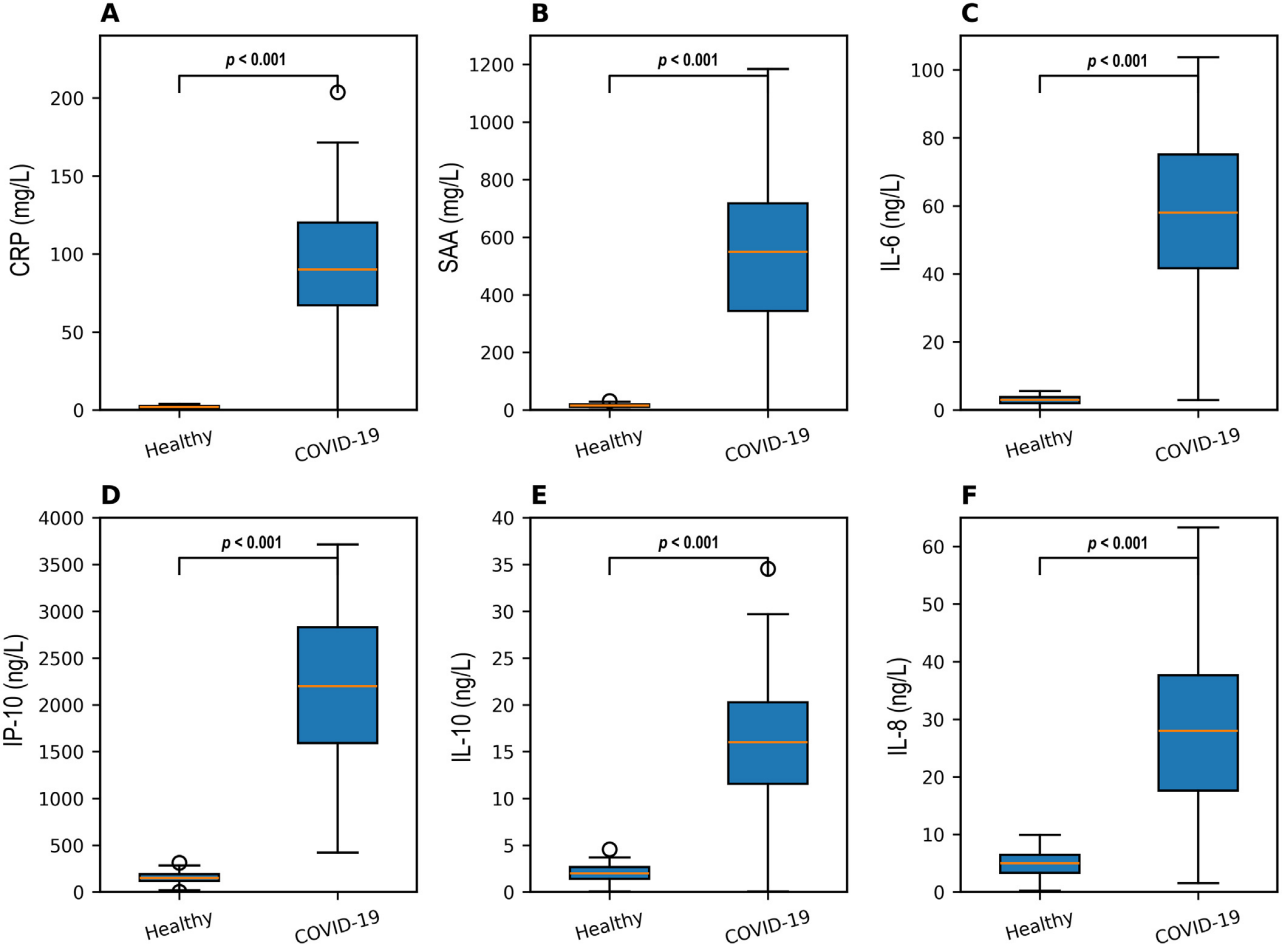
Comparison of inflammatory biomarkers between COVID-19 patients and healthy controls. (A) CRP; (B) SAA; (C) IL-6; (D) IP-10; (E) IL-10; (F) IL-8. Box-and-whisker plots are median, interquartile range, and minimum to maximum. Data are presented as median (interquartile range). Statistical comparisons were performed using the Mann-Whitney *U* test. All comparisons between COVID-19 patients and healthy controls were significant at *p* < 0.001. *Abbreviations*: CRP, C-reactive protein; SAA, serum amyloid A; IL, interleukin; IP-10, interferon-γ-inducible protein-10.

All measured inflammatory biomarkers (IL-6, IL-8, IL-10, IP-10, CRP, SAA) were significantly elevated in COVID-19 patients versus controls (*p* < 0.05; Fig. 3). Spearman correlation analysis revealed strong positive correlations between TOS/OSI and the inflammatory mediators IL-6, IL-10, IP-10, CRP, and SAA (*r*-values 0.597–0.645, all *p* < 0.001). TAS showed weak negative correlations with these markers (Table 2).

**Table 2:** Spearman correlation matrix between oxidative stress and inflammatory markers.

Parameter	TOS	TAS	OSI	CRP	SAA	IL-6	IP-10	IL-10
TOS	—	−0.451*	0.977*	0.631*	0.628*	0.597*	0.420*	0.425*
TAS	—	—	−0.595*	−0.391*	−0.384*	−0.398*	−0.336*	−0.242*
OSI	—	—	—	0.645*	0.638*	0.613*	0.446*	0.435*

*Note*: *p < 0.05.

*Abbreviations*: TOS, total oxidant status; TAS, total antioxidant status; OSI, oxidative stress index; IL, interleukin; IP-10, interferon-γ-inducible protein; CRP, C-reactive protein; SAA, serum amyloid A.

### 3.4 Diagnostic performance and multivariate analysis

**Fig. 4:**
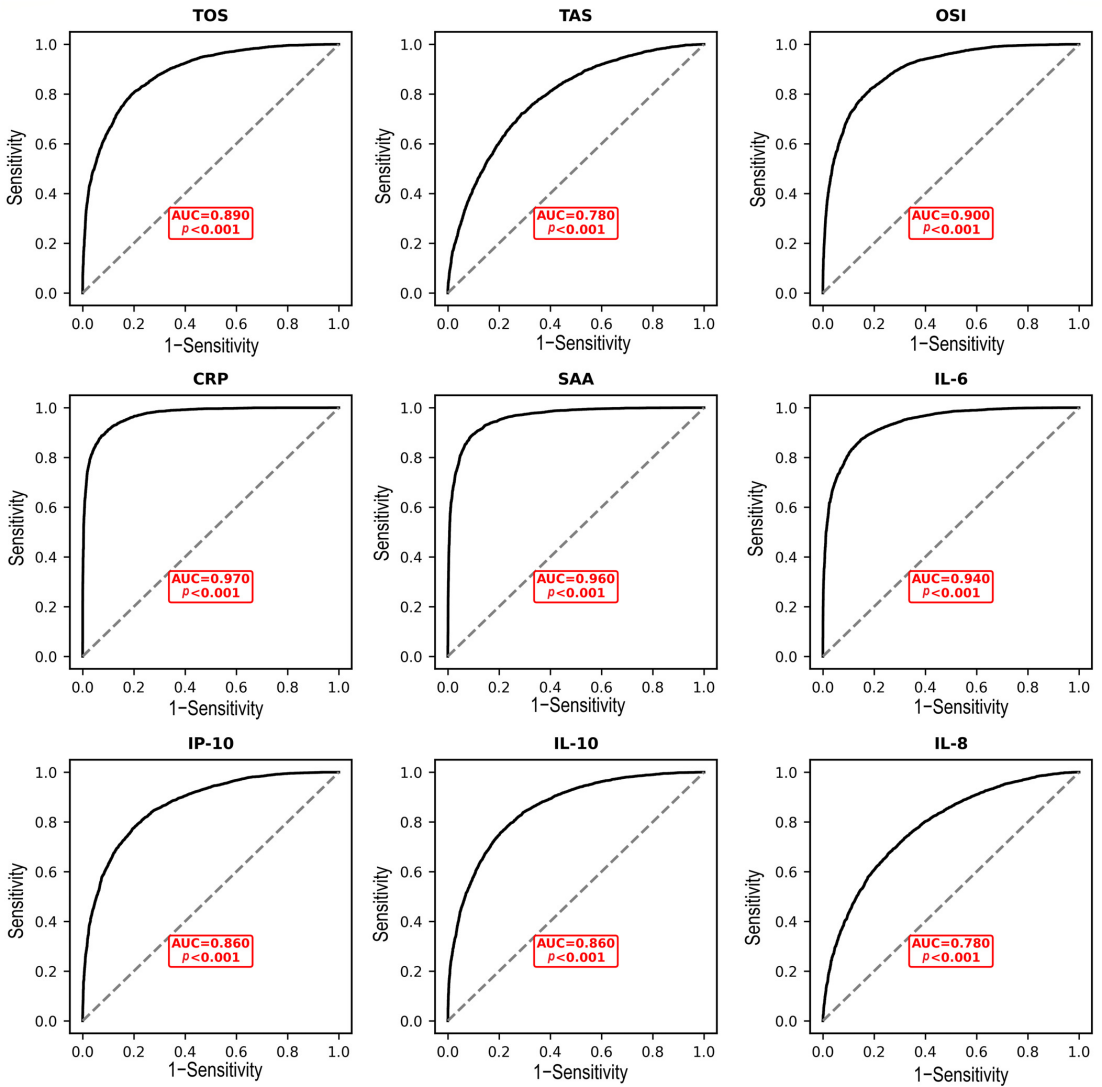
Receiver operating characteristics curves comparing biomarker discrimination of disease severity (severe vs. mild-moderate COVID-19). Receiver operating characteristics curves were compared using the DeLong test. *Abbreviations*: TOS, total oxidant status; TAS, total antioxidant status; OSI, oxidative stress index; CRP, C-reactive protein; SAA, serum amyloid A; IL-6, interleukin-6; IP-10, interferon-γ-inducible protein-10; IL-10, interleukin-10; IL-8, interleukin-8; AUC, area under the curve.

ROC curve analysis demonstrated that OSI had excellent discriminatory power for identifying disease severity (AUC = 0.90; 95% CI: 0.86–0.95; Bootstrap 95% CI: 0.85–0.94), comparable to TOS (AUC = 0.89; 95% CI: 0.84–0.93) and substantially superior to TAS (AUC = 0.78; 95% CI: 0.72–0.85) (Fig. 4, Table 3). The DeLong test confirmed that the AUC of OSI was not significantly different from that of TOS (*p* = 0.12) but was significantly larger than the AUC of TAS (*p* = 0.003). In a multivariate logistic regression model adjusting for age, time from symptom onset, and CCI, a high OSI (above the median) remained a strong independent predictor of severe disease (adjusted odds ratio: 4.8; 95% CI: 1.9–12.1; *p* = 0.001). The inclusion of an interaction term between OSI and age was not statistically significant (*p* = 0.18), indicating that the predictive effect of OSI was consistent across age groups. All VIF values in the final model were below 2.0, indicating no concerning multicollinearity.

**Table 3:** Receiver operating characteristics analysis predicting severe COVID-19.

Biomarker	AUC (95% CI)	Sensitivity (%)	Specificity (%)	PPV (%)	NPV (%)	LR+	LR−	Cut-off
OSI	0.90 (0.86–0.95)	87.5	81.1	82.9	86.2	4.64	0.15	> 3.85
TOS	0.89 (0.84–0.93)	83.9	79.2	80.7	82.5	4.04	0.20	> 1120.5 µmol/L
TAS	0.78 (0.72–0.85)	71.4	73.6	73.3	71.7	2.70	0.39	< 348.2 µmol/L
CRP	0.97 (0.95–0.99)	94.6	92.5	92.9	94.3	12.61	0.06	> 48.0 mg/L

*Abbreviations:* AUC, area under the curve; CI, confidence interval; PPV, positive predictive value; NPV, negative predictive value; LR+, positive likelihood ratio; LR−, negative likelihood ratio; TOS, total oxidant status; TAS, total antioxidant status; OSI, oxidative stress index; CRP, C-reactive protein.

## 4 Discussion

This study provides a comprehensive analysis firmly establishing a pronounced disruption of redox homeostasis in hospitalized COVID-19 patients, the extent of which is directly linked to clinical severity and the accompanying hyperinflammation. Our findings advance the field by demonstrating that the OSI, a composite marker reflecting the net balance between oxidants and antioxidants, is a particularly robust biomarker.

The significantly elevated TOS and depleted TAS in patients align with the known pathophysiology of SARS-CoV-2. The virus induces ROS production through mitochondrial dysfunction and activation of nicotinamide adenine dinucleotide phosphate oxidases in phagocytes.^5^, ^14^ The subsequent depletion of TAS likely represents the consumption of antioxidant reserves in a futile attempt to counter the overwhelming oxidative burst, a phenomenon observed in other severe infections.^9^

The strong, positive correlations we found between TOS/OSI and key drivers of the cytokine storm (e.g., IL-6, IP-10) and acute-phase proteins (CRP, SAA) are mechanistically significant. They provide clinical corroboration for the proposed vicious cycle where ROS can activate NLRP3 inflammasomes to produce IL-6, which in turn stimulates further ROS production in immune and endothelial cells.^6^^,^^7^ This feed-forward loop amplifies tissue damage, coagulopathy, and organ failure. The link between oxidative stress and endothelial dysfunction further explains the thrombo-inflammatory complications characteristic of severe COVID-19.^8^

While TOS measures the offensive burden and TAS the defensive capacity, OSI integrates both into a single, more physiologically relevant metric. Its independent association with disease severity, even after adjustment for a more robust comorbidity measure (Charlson Comorbidity Index) and time from symptom onset, strengthens its candidacy as a valuable tool for risk stratification. Furthermore, our analysis found no significant interaction between OSI and age, suggesting its predictive value is consistent across different age groups an important consideration given the age disparity often seen in COVID-19 severity. This integrative approach represents a novel contribution beyond previous studies that focused on individual oxidative markers.

Our study has limitations. The single-center design may affect generalizability, and while the sample size was adequate for the primary objective (with post-hoc power > 99%), it may limit the precision of estimates for subgroup analyses, as reflected in the width of some confidence intervals. Bootstrap validation provided supporting evidence for the stability of our key estimates. The observational nature precludes causal inference. While we adjusted for time from symptom onset and used a comprehensive comorbidity index, unmeasured confounding factors may persist. Temporal sampling information and treatment exposure variables could provide additional mechanistic insights in future studies.

## 5 Conclusions

We demonstrate that oxidative stress is not merely an epiphenomenon but a central player in the pathophysiology of severe COVID-19, intimately linked with the inflammatory cascade. The OSI is a potent, integrative biomarker for disease severity. Future research should focus on longitudinal studies to assess the dynamic change of these markers in response to therapy and their potential role in predicting long-term post-COVID sequelae. Therapeutic strategies targeting oxidative stress pathways, such as activation of the transcription factor nuclear factor erythroid 2-related factor 2 that may regulate the expression of antioxidant proteins that protect against oxidative damage or mitochondria-specific antioxidants, warrant clinical investigation as adjunctive treatments in acute COVID-19.

## CRediT authorship contribution statement

**Abdisa Tufa Bedada:** Writing – original draft, Methodology, Investigation, Formal analysis, Data curation, Conceptualization. **Tewodros Haile Gebremariam:** Project administration, Investigation, Funding acquisition, Data curation, Conceptualization. **Tsegahun Manyazewal:** Investigation, Funding acquisition, Data curation, Conceptualization. **Yidnekachew Asrat Birhan:** Investigation, Data curation, Conceptualization. **Tesfaye Tolessa Dugul:** Writing – review & editing, Validation, Supervision, Resources, Project administration, Investigation, Funding acquisition, Conceptualization. **Solomon Genet Gebre:** Writing – review & editing, Supervision, Project administration, Investigation, Funding acquisition, Conceptualization. **Dominic-Luc Webb:** Writing – review & editing, Supervision, Resources, Project administration, Methodology, Investigation. **Per Martin Hellström:** Writing – review & editing, Visualization, Validation, Supervision, Project administration, Methodology, Investigation, Funding acquisition, Conceptualization.

## Informed consent

All participants have given their signed informed consent to the research undertaken in the present study.

## Organ donation

Not applicable.

## Ethical statement

The study proposal was approved by the Institutional Review Board of the College of Health Sciences, Addis Ababa University, Ethiopia (Ref. No. 004/21/Biochem), and by the Rational Research Ethics Review Committee of the Ministry of Science and Higher Education, Federal Democratic Republic of Ethiopia (Ref. No. MoSHE/04/246/837/21). Site-level permission to conduct the study was obtained from the Tikur Anbessa Specialized Hospital (TASH), College of Health Sciences, Addis Ababa University. Informed consent was obtained from the study participants (Meeting 01/2021, Protocol 004/21/Biochem). All methods were performed in accordance with the relevant guidelines and regulations, and the study adhered to the Declaration of Helsinki.

## Data Availability Statement

The de-identified datasets sourced from COVID-19 patients and healthy individuals that support the findings of this study are available from the corresponding author upon reasonable request.

## Animal treatment

Not applicable.

## Generative AI

Not applicable.

## Funding

Dr Abdisa Tufa gratefully welcomes the help of Addis Ababa University’s CDT-Africa (no. 2021); Professor Per M. Hellström and Dr. Dominic-Luc Webb, Department of Medical Sciences, Gastroenterology and Hepatology Unit, Uppsala University, Sweden, (no. 5401). DLW gratefully acknowledges support from Selander’s Foundation (2020, 2021) as well as OE and Edla Johansson’s Science Foundation (2017, 2018). DLW and PMH gratefully acknowledges funding from ALF-medel (Uppsala-Örebro region, ALF-899261) and Vetenskapsrådet (2017-02243).

## Declaration of competing interest

All authors declare no competing financial interests or personal relationships that might have appeared to influence the work reported herein.

## Appendix Supplementary materials

Image, application 1
