# Supplementary material for: Oxidative stress index as a potent predictor of disease severity and inflammatory burden in hospitalized COVID-19 patients
Source: Infect Med (Beijing). 2026 May 7;5(2):100261. doi: 10.1016/j.imj.2026.100261 (PMC13199675; doi:10.1016/j.imj.2026.100261)
Supplement: Supplementary file 1 [file mmc1.docx]

# Supplementary Figure S1. Correlation of Oxidative Stress Markers with Inflammatory Mediators

A


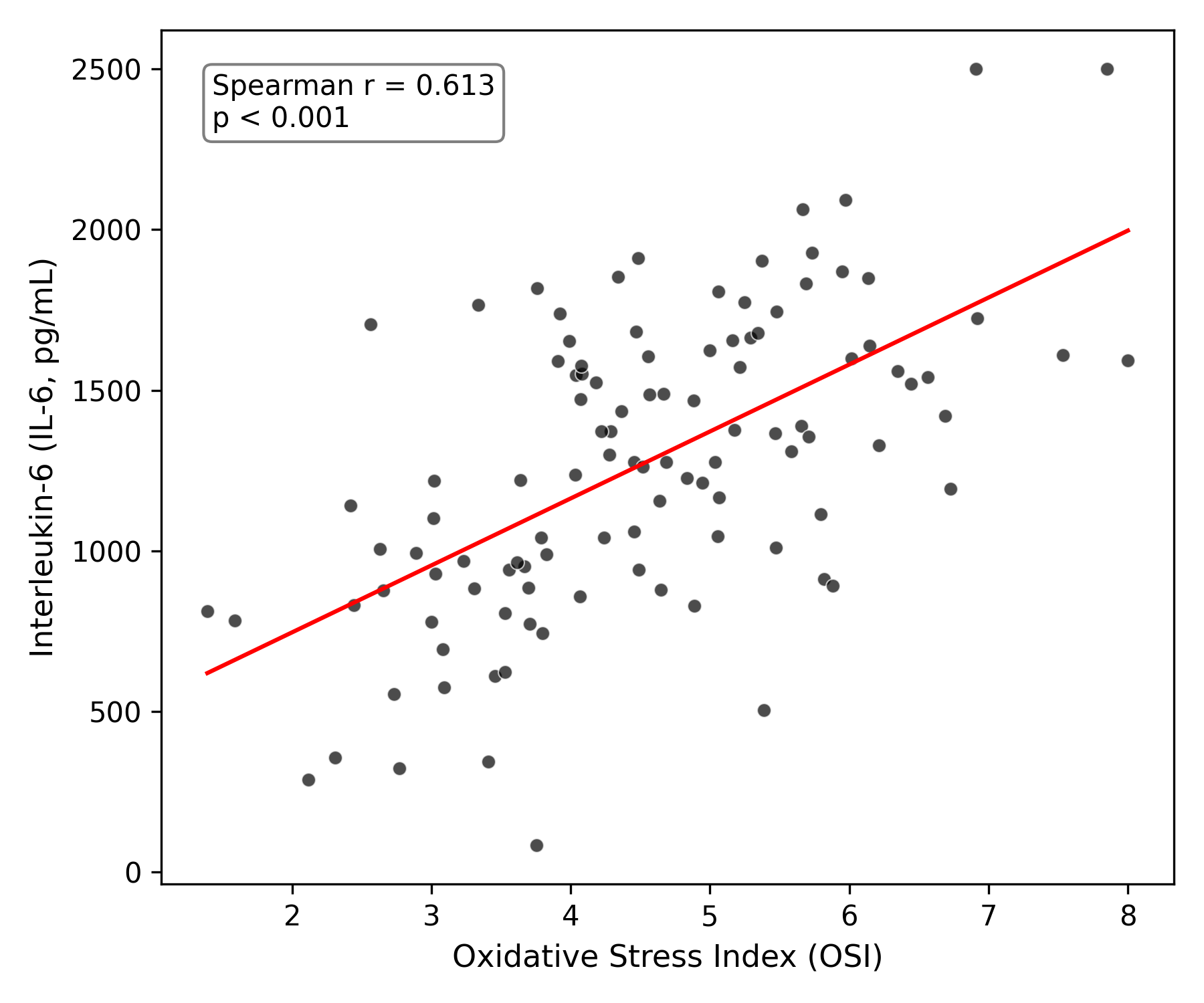


B


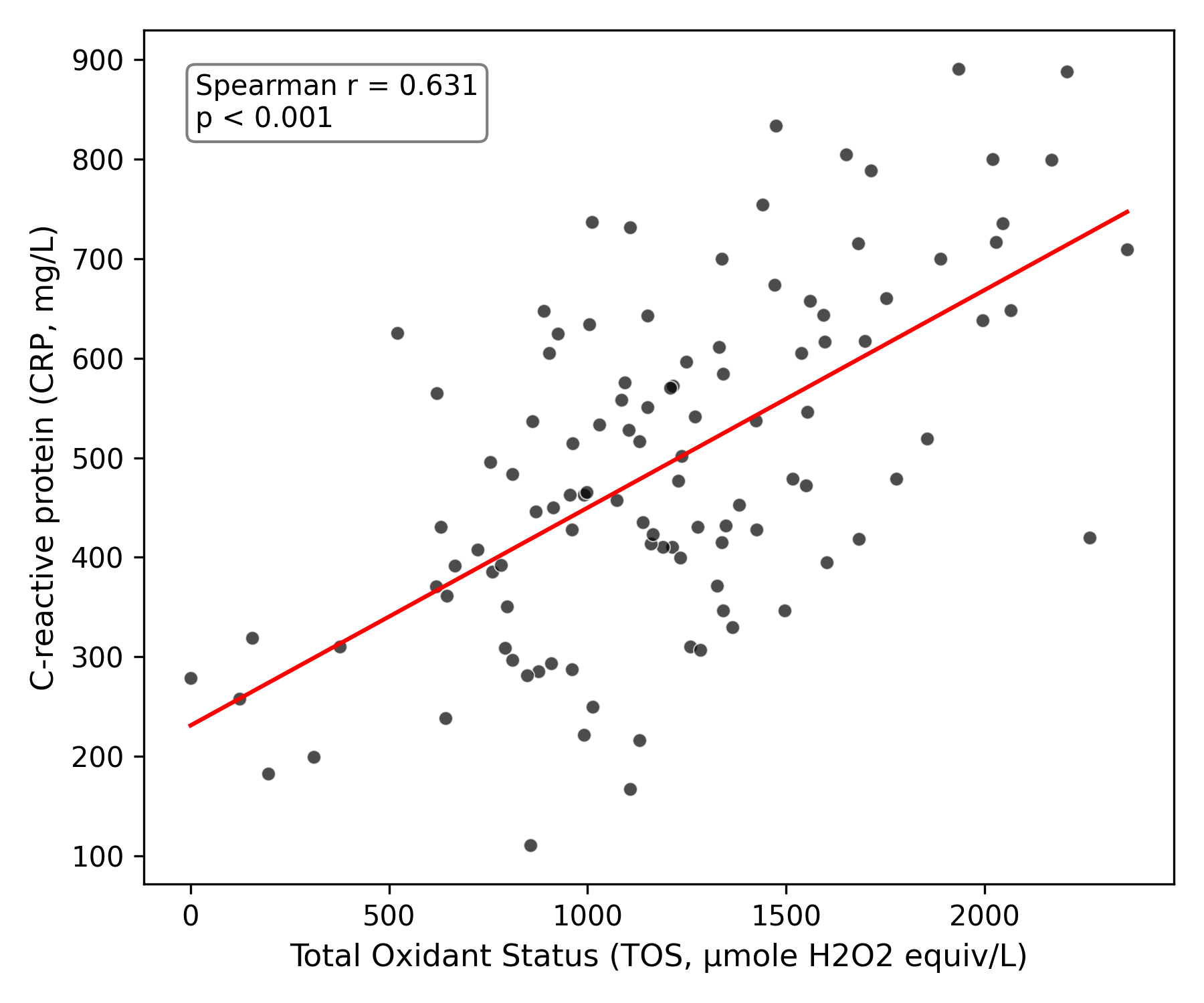


Supplementary Figure S1. Correlation of oxidative stress markers with inflammatory mediators. Scatter plots showing strong positive correlations between (A) Oxidative Stress Index (OSI) and Interleukin-6 (IL-6), and (B) Total Oxidant Status (TOS) and C-reactive protein (CRP) in hospitalized COVID-19 patients (*n* = 109). Spearman's correlation coefficients (*r*) and *p*-values are shown.
